# Observation of stimulated emission from a single Fe-doped AlN triangular fiber at room temperature

**DOI:** 10.1038/srep17979

**Published:** 2015-12-09

**Authors:** Liangbao Jiang, Shifeng Jin, Wenjun Wang, Sibin Zuo, Zhilin Li, Shunchong Wang, Kaixing Zhu, Zhiyi Wei, Xiaolong Chen

**Affiliations:** 1 Research and Development Center for Functional Crystals, Beijing National Laboratory for Condensed Matter Physics, Institute of Physics, Chinese Academy of Sciences, Beijing 100190, China; 2 Beijing National Laboratory for Condensed Matter Physics, Institute of Physics, Chinese Academy of Sciences, Beijing 100190, China; 3 Collaborative Innovation Center of Quantum Matter, Beijing 100190, China

## Abstract

Aluminum nitride (AlN) is a well known wide-band gap semiconductor that has been widely used in fabricating various ultraviolet photo-electronic devices. Herein, we demonstrate that a fiber laser can be achieved in Fe-doped AlN fiber where Fe is the active ion and AlN fiber is used as the gain medium. Fe-doped single crystal AlN fibers with a diameter of 20–50 μm and a length of 0.5–1 mm were preparated successfully. Stimulated emission (peak at about 607 nm and FWHM ~0.2 nm) and a long luminescence lifetime (2.5 ms) were observed in the fibers by a 532nm laser excitation at room temperature. The high quality long AlN fibers are also found to be good optical waveguides. This kind of fiber lasers may possess potential advantages over traditional fiber lasers in enhancing power output and extending laser wavelengths from infrared to visible regime.

Fiber lasers are a class of light sources that exhibit a number of advantages in terms of output power, compactness, and high quality of light beams, and find more and more applications in research, industry and therapeutics as well^1^. The last decades are seeing a dramatic rise in output power from a few watts^2^ in the early 1990s to 30 kW in 2014^3^. The success of the fiber lasers is due in part to the fiber’s geometry, which facilitates the dissipations of heat generation in the fiber core and the efficient enhancement of the energy conversion by cladding-pumping. The gain media where stimulated emission of photons occurs are the rare earths doped silica, germanium oxide, and telluride oxide glasses. Further increase in power output is often retarded by mode instability^4^ and non-linear effects to some extents. In comparison, semiconductor lasers are another class of light sources in which the stimulated emission of photons are released either by an electron-hole plasma process or by an excitonic recombination. Lasing actions have been achieved in many single semiconductor nanowires, such as CdS^5^, ZnO^6^,^7^, GaN^8^, and GaAs^9^ with low pump thresholds. Though these semiconductor materials have better thermal conductivity than glasses, the semiconductors fiber lasers in general have relatively higher noises and lower gains. In this communication, we demonstrate that the simulated emission of photons can be achieved in Fe-doped AlN (Fe:AlN) fibers, which combines the advanges of high efficient transition of electrons in solid state lasers and the high thermal conductivity in wide band-gap semiconductor AlN. Our results may provide a route for further development of fiber lasers with higher power outputs and extension of fiber laser wavelengths from infrared to visible light.

AlN is known as a semiconductor with a wide direct band-gap ~6.2 eV@room temperature (RT)^10^, high thermal conductivity 220–320 W m^−1^ K^−1^ @RT^11^,^12^,^13^,^14^, and chemical inertness, easy doping and environmental friendliness. The combination of these properties makes it an excellent candidate for fabricating deep-UV optoelectronic and high-power, high-frequency electronic devices. For instance, Taniyasu and coworkers^15^ successfully realized the shortest wavelength of luminescence at 210 nm at room temperature from the AlN p-i-n homojunction light-emitting diodes. Recently AlN-based laser diodes have also received intensive research^16^,^17^,^18^,^19^,^20^. Apart from the aforementioned properties, AlN crystallizes in a wurtzite structure and exhibits a strong crystal field. The atomic energy levels of some transition metals, if doped, are expected to split in AlN, just as the case with rare earths in garnets, YVO_4_ and silica. A suitable span between energy levels might be built up, which can be utilized to realize the spontaneous or stimulated photon emissions. In fact, the photoluminescence (PL) in Cu, Mn, Cr, Ti, or Ni-doped AlN^21^,^22^,^23^,^24^ has already been observed. Another aspect deserves being mentioned is that AlN is transparent to a wide wavelength of light from ultraviolet to infrared and exhibits very high refractive index ~2.15^25^. So, in the context of fiber laser, AlN is an attractive material for the gain medium, which may help further increase the power output. However, AlN as a fiber used for fiber lasers has not been reported to date.

The Fe:AlN fibers were grown by vapor-solid process in an induction heating furnace^24^. The detailed description of growth and characterization of Fe:AlN fibers can be found in the supporting information.

The powder X-ray diffraction patterns in Fig. 1a show characteristic peaks with single phase as wurtzite AlN for 0.13 at.% and 0.28 at.% Fe-doped AlN fibers. No other impure peaks were detected within the instrumental resolution, confirming the Fe-doping did not destroy the hexagonal structure of AlN. Figure 1b is the side-view image of Fe:AlN fibers with the average length of about 0.5–1 mm. From the SEM image of Fe:AlN fibers as shown in the inset of Fig. 1b, it can be seen that the Fe:AlN fibers exhibit triangular profiles with diameters ranging from 20 to 50 μm. Figure 1c shows the typical HRTEM image of the Fe:AlN fibers. The spacing of 2.49 Å between adjacent lattice planes corresponds to (002) spacing, indicating [0001] is the growth direction for the Fe:AlN fibers. This [0001] growth direction is also confirmed by the result of selected area electron diffraction (SAED) as shown in the inset of Fig. 1c. These results establish the quality monocrystalline nature of the Fe:AlN fibers. The room temperature excitation and emission spectra of Fe:AlN fibers are shown in Fig. 1d. The excitation spectra exhibit a broad band centered at 495 nm which indicates that there is a strong absorption at this wavelength. The emission spectrum of Fe:AlN fibers excited at 495 nm shows a broad band centered at about 600 nm, showing that Fe:AlN has potential candidate gain medium for fiber lasers considering the difference in emission and excitation wavelengths is not wide since the difference, i.e. the quantum defect, determines the surplus heat in lasing action.

Figure 2a is the far-field image of a representative single Fe:AlN fiber. The far-field image Fig. 2b,c show optically pumped (325 nm, He-Cd laser, 7 mW) emission from a single Fe:AlN fiber (0.13 at.% Fe and 0.28 at.% Fe, respectively). The area I in Fig. 2b,c is the *in-situ* PL under laser excitation, most of which was guided along the fibers and emitted at the fiber end. The orange emission as shown in Fig. 2b,c is very consistent with the emission wavelength measured by Xe lamp as shown in Fig. 1d. The localization of bright emission at the end of the fibers (area II) with relatively weak emission in other regions suggests that strong waveguiding behavior^8^,^26^. Further investigations reveal that all the obtained Fe:AlN fibers have a similar waveguide effect, suggesting that the obtained Fe:AlN fibers can act as good optical waveguide media. These observations demonstrate that they are suitable to realize lasing action.

Figure 3 shows the intensity-dependent PL spectra of a representative 0.28 at.% Fe-doped AlN fiber excited by a 532 nm laser (Nd:YAG) at room temperature. At low excitation intensities, a broad weak emission band appears at about 607 nm which is a little shift compared with the emission at about 600 nm excited by the Xe lamp as shown in Fig. 1d. However, with increasing excitation power density to exceed the threshold (2 mW/μm^2^), a super narrow emission of a single lasing mode (peak at about 607 nm and FWHM ~0.2 nm) occurs. Above the threshold, the integrated emission intensity increases rapidly with the pump power density as shown in Fig. 3 right inset. Apparently, the increment of emission intensity with the excitation power density demonstrates that 0.13 at.% Fe-doped AlN fibers can also realize stimulated emission under a higher intensity excitation, with a threshold power density of around 4.5 mW/μm^2^. However, For 0.13 at.% Fe-doped AlN fibers, the threshold (4.5 mW/μm^2^) is larger than 0.28 at.% Fe-doped, but the lasing intensity is lower as shown in Fig. 3 right inset. The different threshold of Fe:AlN fibers may mainly be attributed to two aspects. Firstly, the fibers themselves are natural Farby-Perot (F-P) cavity to realize stimulated emission or lasing. The threshold of a F-P cavity is inversely proportional to the cavity length (L) by G_th_ ~(2L)^−1^ln(R_1_R_2_)^−1^, where R_1_ and R_2_ are the end facet reflections^27^. According to this relationship, different length and end facet roughness of Fe-doped AlN fibers may cause different thresholds. Secondly, different Fe contents in two samples are also responsible for the variation of the threshold^28^,^29^,^30^. However, we did not observe stimulated emission in undoped AlN fibers even the pump power density reached 20 mW/μm^2^. This indicates that Fe ions play an important role in achieving stimulated emission in AlN. However, in some cases, different PL features were observed in fibers doped with similar Fe contents, as shown in Figure S1(b,c). The reason is not clear at the moment, but we expect that the different PL feature can be attributed to inhomogeneous dopings in AlN fibers. Comparison of the emission intensity at 607 nm of 0.0, 0.13 & 0.28 at.% Fe-doped AlN fibers under different pump fluence was summarized in Table 1.

In addition, for high-quality fibers, single-, and multimode lasing may be achieved along the crystallographic c axis with the two end facets acting as end mirrors forming a F-P cavity. The mode-space Δλ could be calculated by using the expression^31^ Δλ = λ^2^/2 nL, where L is the laser cavity length, n is the refractive index (2.15), and λ is the resonant wavelength (607 nm). For a Fe:AlN fiber with length of 0.5–1 mm, the mode-space between the closest longitudinal modes is expected to be 0.09–0.18 nm. The full width at half maximum of the lasing peak is 0.2 nm as shown in Fig. 3. Therefore, it should exist 1-2 F-P modes for the observed lasing at around 607 nm. This calculated value of longitudinal modes is in good agreement with the experimental results as shown in Fig. 3. However, there should exist multiple transverse modes considering the diameter of the fiber is ~50 μm, though they are not observed in the present study.

Figure 4 shows the luminescence decay curves of the energy level transition from the Fe:AlN fibers at the wavelength of 607 nm. As can be seen, the decay can be well characterized by an exponential function and the luminescence lifetimes of about 2.6 ms (0.13 at.%) and 3.1 ms (0.28 at.%) could be obtained by fitting the decay curve at room temperature. The long lifetimes manifest the high crystal quality of the fibers. It is worth noting that the lifetime in Fe:AlN is much larger than most current laser materials^32^. For example, the lifetime of Mn^3+^ doped GSGG, YSGG, GGG, YGG, YAG is <0.5 μs, 4.7 μs, 2.7 μs, 104.4 μs, 1.11 ms, respectively. Although the mechanism of long lifetime remains unclear at this stage, the long luminescence lifetime provide further evidence that Fe:AlN may be a promising system for high power fiber lasers.

To our best knowledge, the detailed energy levels of Fe^3+^ in hexagonal AlN remain unreported. However, the detailed energy levels of Fe^3+^ in GaN may be taken as a reference for analysis because of the similar lattice constant and crystal field between GaN and AlN^25^. When Al site of AlN was occupied by Fe^3+^, the impact of the N ligand field in the form of a Stark effect cause the d^5^ configuration of Fe^3+^ on Al site to split into the ground state ^6^A_1_(S) and the excited states ^4^T_1_(G), ^4^T_2_(G) and ^4^E(G) as shown in Fig. 3 left inset^33^,^34^,^35^. In GaN, the energy between ^4^T_2_(G) and ^6^A_1_(S) is about 2.009 eV (617 nm), which is smaller than the stimulated emission at 2.043 eV (607 nm). This may be the result of the smaller lattice constant and stronger crystal field of AlN. Therefore, the stimulated emission at 607 nm (2.043 eV) may be attributed to the ^4^T_2_(G)–^6^A_1_(S) transition of Fe^3+^ (left inset in Fig. 3) even though a detailed investigation of energy levels of Fe ion in AlN still needs to be done. In this regard, stimulated emission may also be realized in AlN crystal which doped with other transition metals such as Mn, Co and so on.

In summary, the stimulated emission of photons in Fe:AlN fibers was demonstrated. Stimulated emission (peak at about 607 nm and FWHM ~0.2 nm) and very long luminescence lifetime (2.5 ms) were first observed in the fibers under light excitation at room temperature. The high quality AlN fibers also are good optical waveguides. These results suggest that Fe:AlN have potential applications in high power fiber lasers.

## Additional Information

**How to cite this article**: Jiang, L. *et al.* Observation of stimulated emission from a single Fe-doped AlN triangular fiber at room temperature. *Sci. Rep.* **5**, 17979; doi: 10.1038/srep17979 (2015).

## Supplementary Material

Supplementary Information

## Figures and Tables

**Figure 1:**
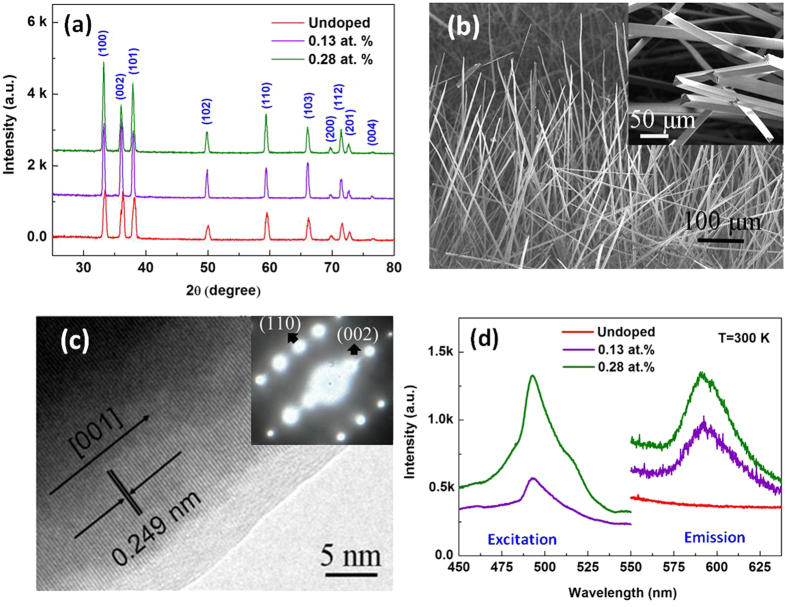
(**a**) XRD pattern of undoped and Fe-doped AlN fibers. (**b**) side view SEM image of Fe:AlN fibers. Inset shows the enlargement of several representative Fe:AlN fibers. (**c**) HRTEM image of an individual Fe:AlN fibers and its corresponding selected area electron diffraction pattern (inset). (**d**) Excitation and emission spectra of Fe:AlN fibers at room temperature (using a 150 W Xe lamp as the excitation source).

**Figure 2:**
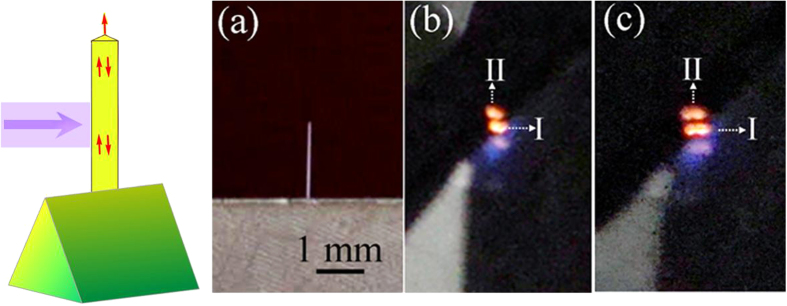
(Left) Schematic of the optically pumped emission from a single Fe:AlN fiber fixing on a wedge-shape sample holder. A 325 nm laser (purple arrow) pumps the fiber, and the fiber emits orange color light. (Right) Far-field image of a representative single Fe:AlN fiber (**a**), and its corresponding emission images for 0.13 at.% Fe doped AlN fiber (**b**), and 0.28 at.% Fe-doped AlN fiber (**c**).

**Figure 3:**
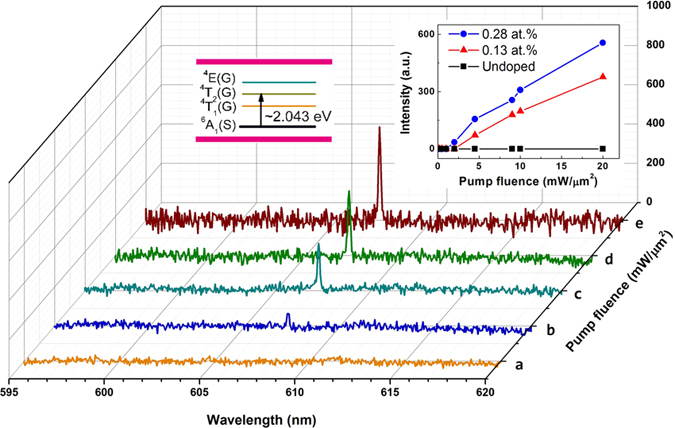
Intensity-dependent PL spectra of 0.28 at.% Fe-doped AlN fiber (a-e corresponding to the pump fluence of 1, 2, 4.5, 9, 20 mW/μm^2^, respectively). (Left inset) the relevant energy levels of Fe ions (3d^5^ electronic configuration); (Right inset) Intensity-dependent PL intensity of 0.0, 0.13 & 0.28 at.% Fe-doped AlN fibers.

**Figure 4:**
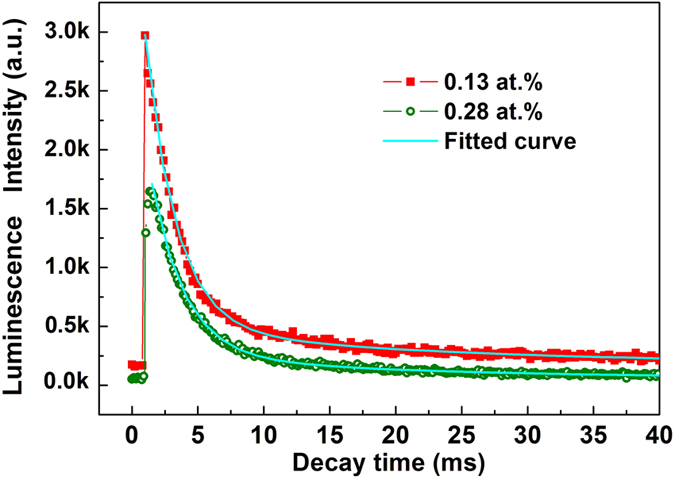
Room-temperature luminescence decay curves of the Fe:AlN fibers.

**Table 1:** Comparison of the emission intensity at 607 nm of 0.0, 0.13 & 0.28 at.% Fe-doped AlN fibers under different pump fluence (unit: mW/μm^2^)

	Emission intensity at 607 nm pumped by different fluence (mW/μm^2^)						
	0.5	1	2	4.5	9	10	20
0.0 at.%	0	0	0	0	0	0	0
0.13at.%	0	0	0	72	179	197	377
0.28 at.%	0	0	35	156	256	309	556

## References

[b1] Nilsson J. & Payne D. N. High-powder fiber lasers. Science 332, 921–922 (2011). 21596979 10.1126/science.1194863

[b2] Minelly J. D. *et al.* High-power diode-pumped Nd^3+^ fiber laser. Proc. SPIE 2131, 292 (1994).

[b3] *Lockheed Martin demonstrates weapons grade high power fiber laser.* (2014) Available at: http://www.lockheedmartin.com/us/news/press-releases/2014/january/140128-mst-lockheed-martin-demonstrates-weapons-grade-high-power-fiber-laser.html. (Accessed: 20th March 2015).

[b4] Jauregui C., Limpert J. & Andreas T. High-power fiber lasers. Nat. Photonics 7, 861–867 (2013).

[b5] Duan X. F., Huang Y., Agarwal R. & Lieber C. M. Single-nanowire electrically driven lasers. Nature 421, 241–245 (2003). 12529637 10.1038/nature01353

[b6] Huang M. H. *et al.* Room-temperature ultraviolet nanowire nanolasers. Science 292, 1897–1899 (2001). 11397941 10.1126/science.1060367

[b7] Xu C. X. *et al.* Whispering-gallery mode lasing in ZnO microcavities. Laser Photonics Rev. 8, 469–494 (2014).

[b8] Johnson J. C. *et al.* Single Gallium nitride nanowire lasers. Nat. Mater. 1, 106–110 (2002). 12618824 10.1038/nmat728

[b9] Saxena D. *et al.* Optically pumped room-temperature GaAs nanowire lasers. Nat. Photonics 7, 963–968 (2013).

[b10] Yim W. M. *et al.* Epitaxially grown AlN and its optical band gap. J. Appl. Phys. 44, 292 (1973).

[b11] Slack G. A. Nonmetallic crystals with high thermal conductivity. J. Phys. Chem. Solids 34, 321–335 (1973).

[b12] Slack G. A. & McNelly T. F. Growth of high purity AlN crystals. J. Cryst. Growth 34, 263–279 (1976).

[b13] Slack G. A. & McNelly T. F. AlN single crystals. J. Cryst. Growth 42, 560–563 (1977).

[b14] Slack G. A., Tanzilli R. A., Pohl R. O. & Vandersande J. W. The intrinsic thermal conductivity of AlN. J. Phys. Chem. Solids 48, 641–647 (1987).

[b15] Taniyasu Y., Kasu M. & Makimoto T. An aluminium nitride light-emitting diode with a wavelength of 210 nanometres. Nature 441, 325 (2006). 16710416 10.1038/nature04760

[b16] Krukowski S. *et al.* Blue and UV semiconductor lasers. Acta Phys. Pol. B 37, 1265–1312 (2006).

[b17] Akasaki I. Key inventions in the history of nitride-based blue LED and LD. J. Cryst. Growth 300, 2–10 (2007).

[b18] Yoshida H., Yamashita Y., Kuwabara M. & Kan H. Demonstration of an ultraviolet 336 nm AlGaN multiple-quantum-well laser diode. Appl. Phys. Lett. 93, 241106 (2008).

[b19] Onuma T., Hazu K., Uedono A., Sota T. & Chichibu S. F. Identification of extremely radiative nature of AlN by time-resolved photoluminescence. Appl. Phys. Lett. 96, 061906 (2010).

[b20] Oto T., Banal R. G., Kataoka K., Funato M. & Kawakami Y. 100 mW deep-ultraviolet emission from aluminium-nitride-based quantum wells pumped by an electron beam. Nat. Photonics 4, 767–771 (2010).

[b21] Martin A. L. *et al.* Visible emission from amorphous AlN thin-film phosphors with Cu, Mn, or Cr. J. Vac. Sci. Technol. A 19, 1894–1897 (2001).

[b22] Maqbool M., Main K. & Kordesch M. Titanium-doped sputter-deposited AlN infrared whispering gallery mode microlaser on optical fibers. Opt. Lett. 35, 3637–3639 (2010). 21042375 10.1364/OL.35.003637

[b23] Li H., Chen X. L., Song B., Bao H. Q. & Wang W. J. Copper-doped AlN polycrystalline powders: A class of room-temperature ferromagnetic materials. Solid State Commun. 151, 499 (2011).

[b24] Jiang L. B. *et al.* Role of Ni in the controlled growth of single crystal AlN triangular microfibers: Morphology evolvement, growth kinetics and photoluminescence. J. Cryst. Growth 318, 1089 (2011).

[b25] Levinshtein M. E., Rumyantsev S. L. & Shur M. S. Properties of Advanced Semiconductor Materials: GaN, AIN, InN, BN, SiC, SiGe. John Wiley & Sons, (2001).

[b26] Pan A. L. *et al.* High-quality alloyed CdS_x_Se_1−x_ fibers as waveguides with tunable stimulated emission. J. Phys. Chem. B 110, 22313–22317 (2006). 17091969 10.1021/jp064664s

[b27] Han X. *et al.* Ultraviolet lasing and time-resolved photoluminescence of well-aligned ZnO nanorod arrays. Appl. Phys. Lett. 86, 223106 (2005).

[b28] Ikesue A., Kamata K. & Yoshida K. Effects of Neodymium Concentration on Optical Characteristics of Polycrystalline Nd:YAG Laser Materials. J. Am. Ceram. Soc. 79, 1921–1926 (1996).

[b29] Diettrich J. C., McKinnie I. T. & Warrington D. M. The influence of active ion concentration and crystal parameters on pulsed Cr:YAG laser performance. Opt. Commun. 167, 133–140 (1999).

[b30] Jackson S. D. & Mossman S. Diode-cladding-pumped Yb^3+^, Ho^3+^-doped silica fiber laser operating at 2.1 μm. Appl. Optics. 42, 3546–3549 (2003). 10.1364/ao.42.003546 12833958

[b31] Hsu H. C., Wu C. Y. & Hsieh W. F. Stimulated Emission and Lasing of Random-Growth Oriented ZnO Nanowires. J. Appl. Phys. 97, 064315 (2005).

[b32] Kück S. Laser-related spectroscopy of ion-doped crystals for tunable solid-state lasers. Appl. Phys. B 72, 515–562 (2001).

[b33] Heitz R. *et al.* Excited states of Fe^3+^ in GaN. Phys. Rev. B 55, 4382–4387 (1997).

[b34] Malguth E. *et al.* Structural and electronic properties of Fe^3+^ and Fe^2+^ centers in GaN from optical and EPR experiments. Phys. Rev. B 74, 165202 (2006).

[b35] Fujimura R., Kitazaki S., Shimura T. & Kuroda K. Light-induced absorption change in Fe-doped GaN. Opt. Commun. 282, 2174–2177 (2009).

