# Supplementary material for: Observation of stimulated emission from a single Fe-doped AlN triangular fiber at room temperature
Source: Sci Rep. 2015 Dec 9;5:17979. doi: 10.1038/srep17979 (PMC4673607; doi:10.1038/srep17979)
Supplement: Supplementary Information [file srep17979-s1.doc]

**Supporting information for:**

Observation of stimulated emission from a single Fe-doped AlN triangular fiber at room temperature

Liangbao Jiang,^1^ Shifeng Jin,^1^ Wenjun Wang*,^1^ Sibin Zuo,^1^ Zhilin Li,^1^ Shunchong Wang,^1^ Kaixing Zhu,^1^ Zhiyi Wei^2^, and Xiaolong Chen*,^1,3^

1. Research and Development Center for Functional Crystals, Beijing National Laboratory for Condensed Matter Physics, Institute of Physics, Chinese Academy of Sciences, Beijing 100190, China
2. Beijing National Laboratory for Condensed Matter Physics, Institute of Physics, Chinese Academy of Sciences, Beijing 100190, China
3. Collaborative Innovation Center of Quantum Matter, Beijing 100190, China

**1. Growth of Fe-doped AlN fibers and characterizations.**

1.1 Growth of Fe-doped AlN fibers

The Fe-doped AlN (Fe:AlN) fibers were grown by vapor-solid process in an induction heating furnace. AlN (99.99%) and Fe (99.99%) powders were selected as reacted sources. In a typical run, the reactant sources were mixed in an agate mortar, followed by putting into TaC crucible, then TaC crucible was loaded into a graphite crucible. At last, the graphite crucible was placed inside the induction heating furnace. The schematic diagram for the furnace could be found in Ref. S1. After the furnace was evacuated to about 10^-5^ Torr, 0.6 atm N_2_ was introduced into the furnace as the nitrogen source. Then, the crucible was heated to about 1700℃ and kept for 3h. Finally, it was cooled down to room temperature naturally and the products were densely grown on the surface of TaC cover. The doping concentration of Fe was controlled by the mole ratio of AlN and Fe in the source material. The undoped AlN samples that had undergone the same process were served as reference.

1.2 Characterization of Fe:AlN fibers

The as grown products were characterized by X-ray diffractometor (X’Pert ProMRD) with Cu Kα radiation at 40 kV and 40 mA, scanning electron microscope (SEM, FEI, XL-30) and a high resolution transmission electron microscope (HRTEM, JEOL JEM-2010). Inductively coupled plasma-atomic emission spectrometry (ICP-AES) was used to determine the doping concentration of Fe in the as-prepared sample. The excitation and emission spectrum measurements were performed using a Hitachi F-7000 spectrofluorometer with a Xe lamp as the excitation source. The optical waveguide properties were investigated by a continuous He-Cd laser (325 nm, 7 mW). The Fe:AlN fiber was fixed on a wedge-shaped aluminum stage. A color charge coupled device (CCD) camera was used for collecting the far-field optical image of the excited fibers. All measurements were performed at room temperature.

**2. Details description of PL measurement**

The stimulated emission were achieved by a Nd:YAG laser (532 nm). The pumping beam was directed obliquely to the microscope objective (50×, NA=0.5) and focused to one about 2 μm spot which illuminated on the end face of the samples. The emission light of the sample was collected by the same objective and imaged by a CCD camera, with an expected spectral resolution of ~0.02 nm. Time-correlated single photon counting technique was used to detect the luminescence lifetime. The experimental setup for laser excitation and emission probe is shown in Figure S1(a). The sample was pumped with 532 nm Nd:YAG laser. At first, the laser beam was expanded to improve its quality. Then, the expanded laser beam was reflected by four mirrors and directed obliquely to the microscope objective and focused on the sample. The PL was collected by the same objective and pass through a holographic filter and a slit before it was dispersed by a diffraction grating. At last, the PL was detected using a CCD camera.

Figure S1 (b)-(d) shows the intensity-dependent PL spectra of Fe:AlN triangular fibers with different Fe contents. It can be seen that all samples exhibit stimulated emission when the excitation power density exceed the threshold. However, some cases even with similar Fe contents, such as Figure S1 (b) and (c) also exhibit different PL feature. This shows the fact that the doping in AlN fibers may be inhomogeneous.


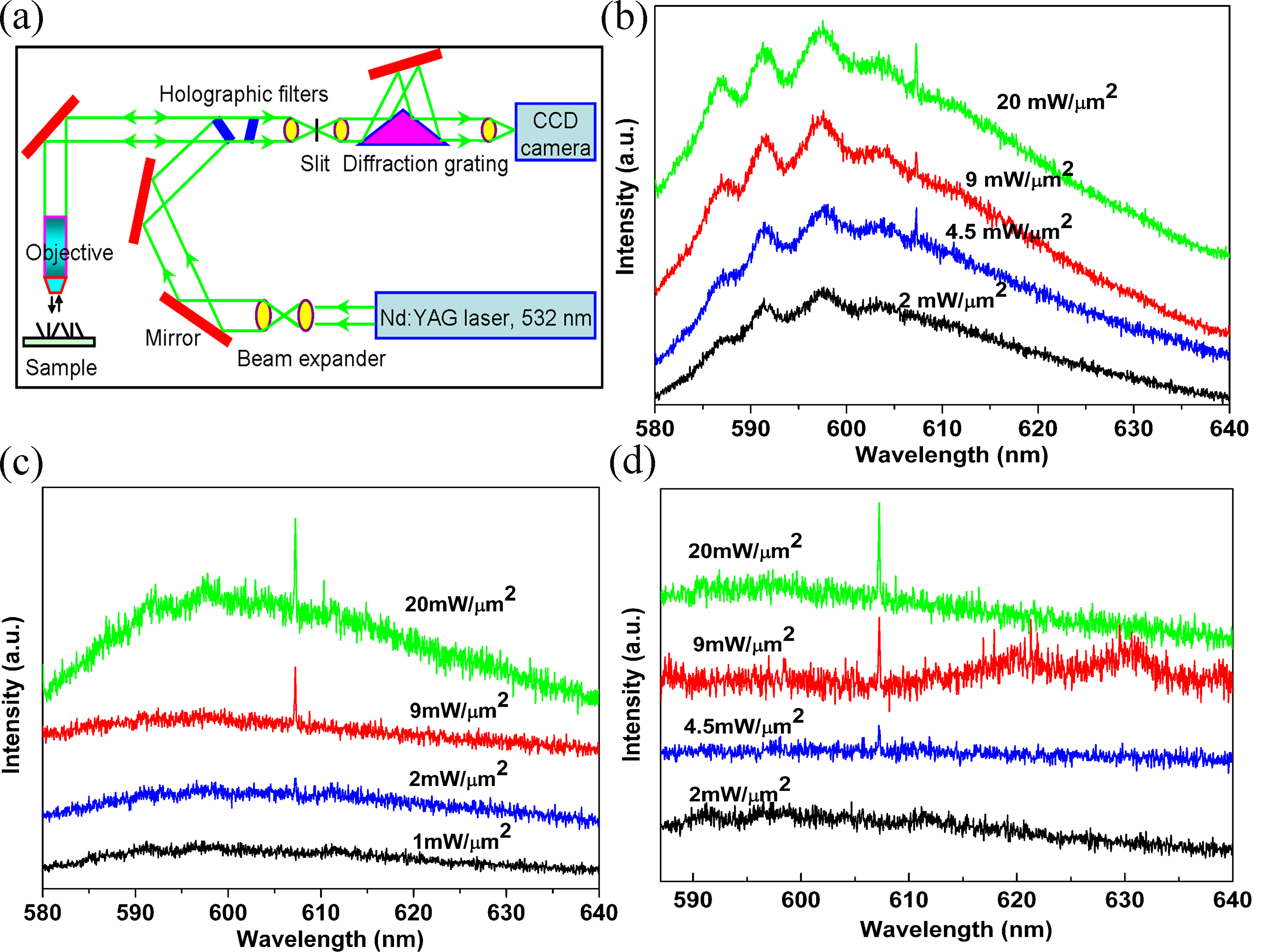


Figure S1 (a) Schematic of the instrumental setup for stimulated emission experiments. (b) Intensity-dependent PL spectra of 0.35 at.% Fe-doped AlN fibers. (c) Intensity-dependent PL spectra of 0.31 at.% Fe-doped AlN fibers. (d) Intensity-dependent PL spectra of 0.19 at.% Fe-doped AlN fibers.

Reference:

[S1] Chen, X. L. Growth of bulk GaN single crystals by flux method. *Sci. Technol. Adv. Mater.* **6**, 766-771 (2005).
